# Laparoscopic versus open resection for gastric gastrointestinal stromal tumors: an updated systematic review and meta-analysis

**DOI:** 10.1186/1477-7819-12-206

**Published:** 2014-07-14

**Authors:** Qi-Long Chen, Yu Pan, Jia-Qin Cai, Di Wu, Ke Chen, Yi-Ping Mou

**Affiliations:** 1 Department of General Surgery, Sir Run Run Shaw Hospital, School of Medicine, Zhejiang University, 3 East Qingchun Road, 310016 Hangzhou, Zhejiang province, China

**Keywords:** Complications, Gastrectomy, Gastrointestinal stromal tumor, Laparoscopy, Meta-analysis

## Abstract

**Background:**

In past decades, laparoscopic surgery has been introduced for the treatment of gastrointestinal stromal tumors (GISTs). Recently, additional studies comparing laparoscopic versus open surgery for gastric GISTs have been published, and an updated meta-analysis of this subject is necessary.

**Methods:**

A systematic search was conducted in PubMed, Embase, Cochrane Library, and Web of Science. Comparative studies of laparoscopic and open surgery for gastric GISTs published before June 2014 were identified from databases. The Newcastle-Ottawa Quality Assessment Scale was used to perform quality assessment and original data were extracted. The statistical software STATA (version 12.0) was used for the meta-analysis.

**Results:**

Finally, 22 studies, including a total of 1,166 cases, meet the inclusion criteria for meta-analysis. The operation time was similar between laparoscopic and open surgery. Compared to open surgery, laparoscopic resection was associated withless blood loss (WMD = -58.91 ml; 95% CI, -84.60 to -33.22 ml; *P* <0.01); earlier time to flatus (WMD = -1.31 d; 95% CI, -1.56 to -1.06, *P* <0.01) and oral diet (WMD = -1.75 d; 95% CI, -2.12 to -1.39; *P* <0.01); shorter hospital stay (WMD = -3.68 d; 95% CI, -4.47 to -2.88; *P* <0.01); and decreased overall complications (relative risk = 0.57; 95% CI, 0.37 to 0.89; *P* = 0.01). For long-term outcomes, there were no significant differences between two surgical procedures on recurrence.

**Conclusion:**

Laparoscopic surgery for gastric GISTs is acceptable for selective patients with better short-term outcomes compared with open surgery. The long-term survival situation of patients mainly depends on the nature of tumor itself, and laparoscopic surgery was not associated with worse oncological outcomes.

## Background

Gastrointestinal stromal tumors (GISTs) are the most common mesenchymal tumor in the gastrointestinal tract and are often characterized by cellular markers, such as CD117 (a c-kit gene proto-oncogene product) and CD34 (a human progenitor cell antigen) [1-3]. GISTs, which frequently occur in the stomach and small intestine [2], have malignant potential, and recurrence of GISTs often occurs at the peritoneal surface or liver [4]. Targeted therapies have been developed for GISTs, but surgical resection remains the optimal initial treatment approach for primary GISTs with no evidence of metastasis. The surgical principles of gastrointestinal stromal tumor comprise *en bloc* resection (R0 resection) with avoidance of rupture, which may result in peritoneal seeding. In addition, lymphadenectomy is not indicated in GISTs because of a very low propensity for lymph node metastases [5].

With the development of minimally invasive surgical approaches, laparoscopic surgery (LAP) for gastrointestinal stromal tumors has evolved rapidly over the past decades. Various types of laparoscopic approaches for GISTs have been performed in a few specialized centers, including wedge resection of the stomach, intragastric tumor resection, and combined endoscopic-laparoscopic resection [6-9]. Owing to the technique difficulty and relative rarity of GISTs, there is few study of large scale of patients reporting the short- and long-term results for LAP for GISTs compared with open surgery (OPEN). To address these issues, our team conducted the following meta-analysis to compare short-term and long-term results of patients undergoing LAP.

## Methods

### Search strategy

A systematic search was conducted in PubMed, Embase, Cochrane Library, and Web of Science to identify articles published up to June 2014. The search terms included ‘gastrointestinal stromal tumor’, ‘GIST’, ‘laparoscop*’, ‘gastrectomy’ and “gastric resection’. A personal search was also performed with reference lists of the retrieved relevant articles and reviews to identify additional trials and ensure that all the potential studies were included. The language of the articles was limited to English and Chinese according to the reviewers’ language competence.

### Study selection

The inclusion criteria were as follows: comparative, peer-reviewed studies of LAP versus OPEN for GISTs for which the full text of the article was available. If two or more studies from the same institution, the most recent study or that including informative data was selected unless the reports were from different time periods. We excluded studies including: GISTs out of the stomach; complicated with mixed disease, such as gastric cancer; studies in which fewer than two relevant indexes were reported, or where it was difficult to calculate these from the results; and studies where the measured outcomes were not clearly presented in the literature.

### Data extraction and quality assessment

Two researchers independently extracted the data and disagreement was resolved through discussion. Extracted data included author, study period, geographical region, number of patients, operation time, blood loss, time to flatus, time to oral intake, length of hospital stay, morbidity, mortality, and long-term outcomes. The Newcastle-Ottawa Quality Assessment Scale (NOS) was used as an assessment tool. This scale varies from zero to nine stars: studies with a score equal to or higher than six were considered methodologically sound.

### Outcome definition and Statistical analysis

Postoperative complications were classified as systematic complications (cardiovascular, respiratory or metabolic events; nonsurgical infections; deep venous thrombosis; and pulmonary embolism) or surgical complications (any anastomotic leakage or fistula, any complication that required reoperation, intra-abdominal collections, wound complications, bleeding events, pancreatitis, ileus, delayed gastric emptying, and anastomotic stricture). This classification system is based on the Memorial Sloan-Kettering Cancer Center complication reporting system [10]. Continuous variables were assessed using weighted mean difference (WMD), and dichotomous variables were analyzed using the risk ratio (RR). If the study provided medians and ranges instead of means and standard deviations, we estimated the means and standard deviations as described by Hozo *et al*. [11]. Statistical heterogeneity was evaluated by the Higgins *I*^2^ statistic [12]. Based on method reported by DerSimonian and Laird [13], substantial significance was set when P < 0.10 and a random effect model was used. We hypothesized the outcomes of the comparison may be affected by the uneven distribution of the surgical types between the LAP and OPEN groups, especially by the relatively larger proportion of extended surgeries performed in the OPEN group. Thus, we performed a subgroup analysis of patients who underwent wedge resection in the two groups to eliminate the bias from the surgical type selection. We also conducted a subgroup analysis of studies that had comparable tumor size or risk index proposed by Fletcher *et al*. [3], which may have an impact on the operative outcomes. The potential publication bias based on the postoperative complications was assessed Begg’s test and funnel plots. Data analyses were performed using STATA (version 12.0). *P* <0.05 was considered statistically significant.

## Results

### Studies selected

**Figure 1:**
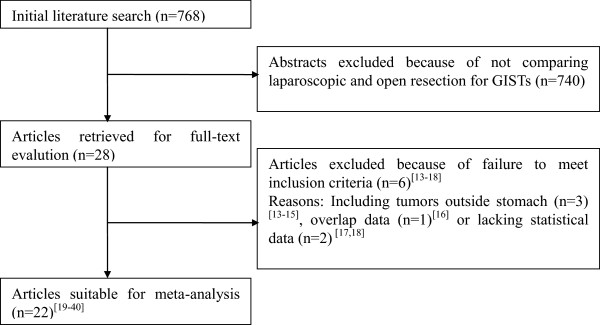
**Flow chart of literature search strategies.** GIST, gastrointestinal stromal tumor.

By the initial search, 768 potentially relevant articles were identified. After the titles and abstracts were reviewed, papers without comparison of LAP and OPEN were excluded, which left 28 comparative studies. An additional six [14-19] studies did not meet the inclusion criteria and were excluded. In total, 22 observational studies were obtained [20-41], all of which were accessible in full-text format. Twenty-one studies were published in English and one in Chinese. A flow chart of the search strategies, which contains reasons for exclusion, is presented in Figure 1.

### Characteristics and quality of studies

A total of 1,166 patients were included in the analysis with 574 undergoing LAP (49.2%) and 592 undergoing OPEN (50.8%). They represented an international experience, with data included from 10 different countries or regions (six Japan, four United States, four China, two Korea, one United Kingdom, one Italy, one Belgium, one Austria, one Singapore and one Taiwan). According to the NOS, one out of the 22 observational studies got six stars, six articles got seven stars, eight articles got eight stars and the remaining seven got nine stars. Overall, all studies were evaluated as being moderate to high quality. The characteristics and methodological quality assessment scores of the included studies are summarized in Table 1.

**Table 1:** Summary of studies included in the meta-analysis

**Author**	**Region**	**Study design**	**Year**	**Study period**	**Sample size**		**Conversion (%)**	**Follow-up (month)**		**Recurrence**		**Quality scores**
					**LAP**	**OPEN**		**LAP**	**OPEN**	**LAP**	**OPEN**	
Shimizu *et al*. [20]	Japan	OCS (R)	2002	1986-2000	11	8	0	NR	NR	NR	NR	7
Matthews *et al*. [21]	USA	OCS (R)	2002	1994-2000	21	12	NR	20	18	1	1	7
Ishikawa *et al*. [22]	Japan	OCS (R)	2006	1993-2004	14	7	NR	60 (5 to 119)	61 (3 to 130)	2	1	8
Mochizuki *et al*. [23]	Japan	OCS (R)	2006	2000-2004	12	10	NR	26 (6 to 53)	NR	0	0	8
Nishimura *et al*. [24]	Japan	OCS (R)	2007	1993-2004	39	28	2.6	18.9 (2.6 to 96.4)	31.2 (4.4 to 121.9)	1	4	9
Pitsinis *et al*. [25]	UK	OCS (P)	2007	2004-2006	6	7	NR	9	9	0	0	6
Catena *et al*. [26]	Italy	OCS (P)	2008	1995-2006	21	25	NR	35 (5 to 58)	91 (80 to 136)	0	1	9
Silberhumer *et al*. [27]	Austria	OCS (R)	2009	1998-2006	22	41	18.2	30 ± 20	41 ± 31	0	4	8
Goh *et al*. [28]	Singapore	OCS (R)	2010	2001-2009	14	39	7.1	8 (3 to 60)	21(2 to 72)	0	2	7
Karakousis *et al*. [29]	USA	OCS (P)	2011	1998-2009	40	40	22.5	28 (0.3 to 70)	43 (0.1 to 139)	1	1	9
Dai *et al*. [30]	China	OCS (R)	2011	2000-2009	18	30	NR	78	64	2	3	9
De Vogelaere *et al*. [31]	Belgium	OCS (P)	2012	1997-2011	37	16	NR	83 (2 to 163)	71 (0.3 to 199)	0	6	8
Melstrom *et al*. [32]	USA	OCS (P)	2012	1999-2008	17	29	5.9	32	59	0	4	7
Lee *et al*. [33]	Korea	OCS (R)	2011	2001-2008	50	50	2	21.1 (0 to 64)	22.3 (0 to 93)	0	0	9
Wan *et al*. [34]	China	OCS (R)	2012	2004-2011	68	88	NR	29 (4 to 89)	36 (4 to 90)	3	4	9
Pucci *et al*. [35]	USA	OCS (P)	2012	2002-2012	57	47	1.8	NR	NR	NR		7
Kim *et al*. [36]	Korea	OCS (R)	2012	1998-2011	24	14	NR	62.6 (8.9 to 164.4)	58.3 (18.8 to 123.2)	1	3	7
Shu *et al*. [37]	China	OCS (R)	2013	2010-2012	15	21	NR	NR	NR	N	NR	8
Lee *et al*. [38]	Taiwan	OCS (R)	2013	2007-2009	30	32	NR	NR	NR	NR	NR	8
Kasetsermwiriya *et al*. [39]	Japan	OCS (R)	2014	1988-2011	23	10	NR	46 (2 to 168)	19 (1 to 275)	0	1	8
Lin *et al*. [40]	China	OCS (R)	2014	2007-2012	23	23	4.3	34 (6 to 78)		2	3	9
Takahashi *et al*. [41]	Japan	OCS (R)	2014	1995-2011	12	15	25	57 (7 to 120)	69 (13 to 154)	1	2	8

LAP, laparoscopic surgery; NR, not reported; OCS, observational clinical study; OPEN, open surgery; P, prospectively collected data; R, retrospectively collected data.

### Comparison of operative outcomes

**Figure 2:**
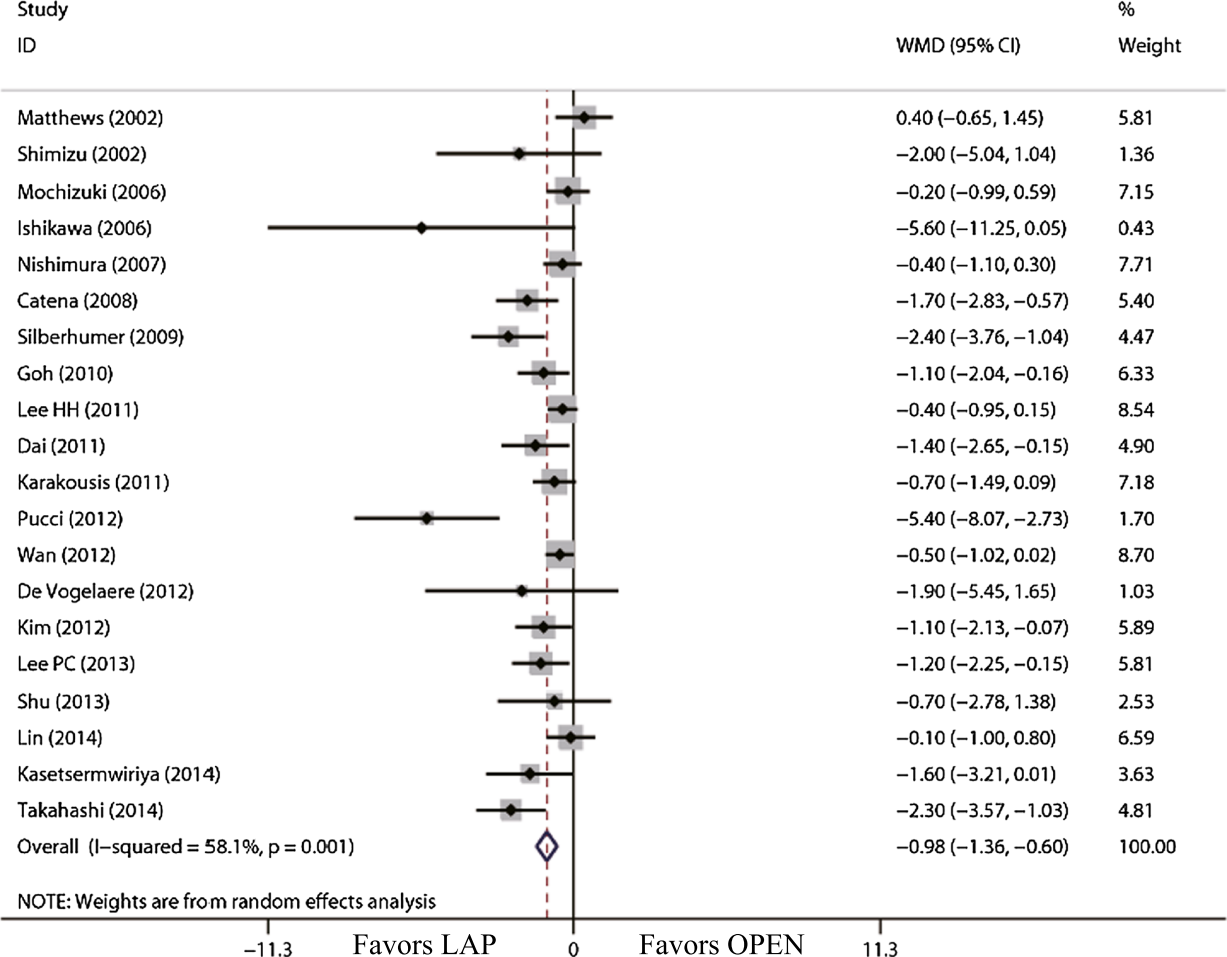
Meta-analysis of the pooled data: tumor size (cm).

**Figure 3:**
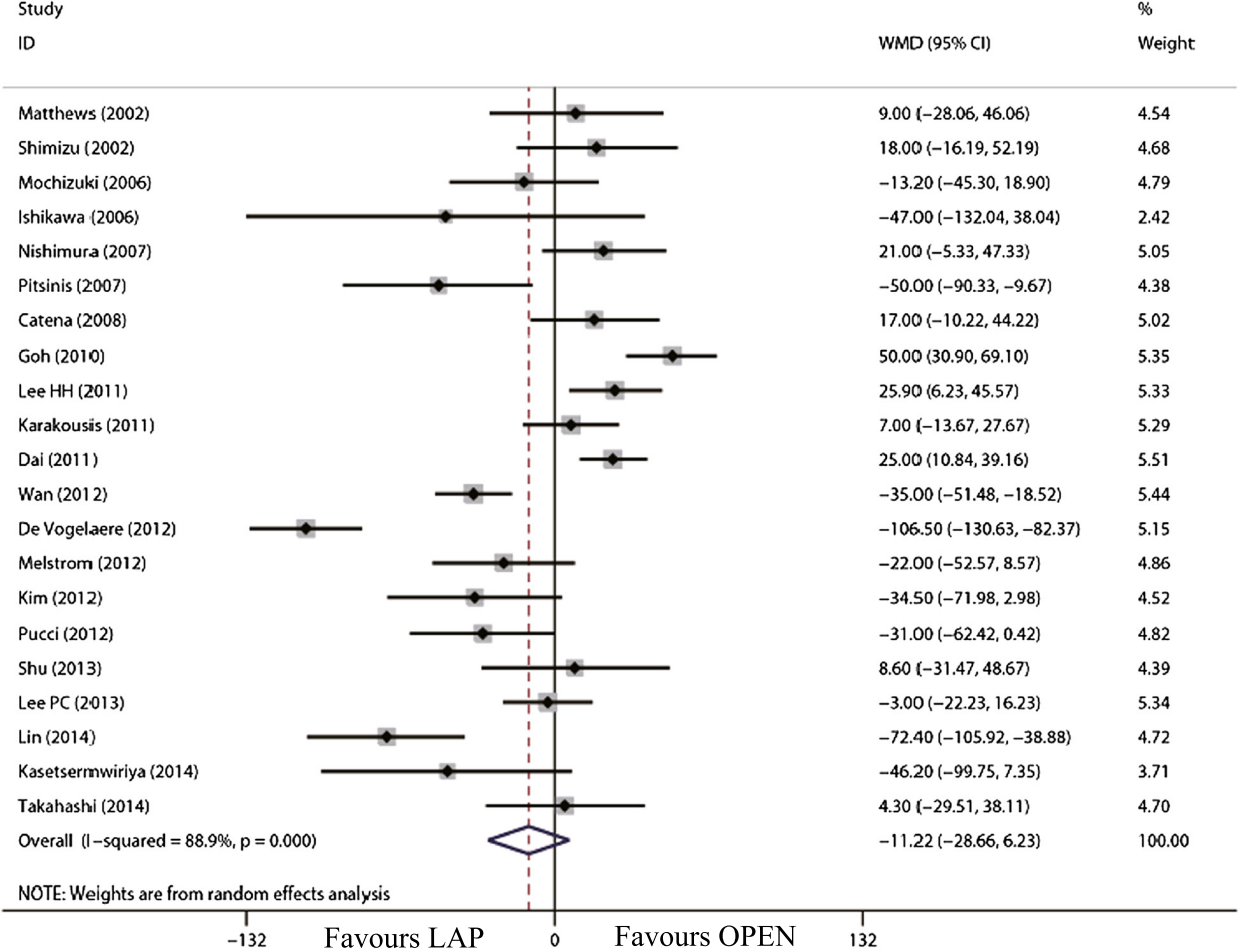
Meta-analysis of the pooled data: operation time (minutes).

**Figure 4:**
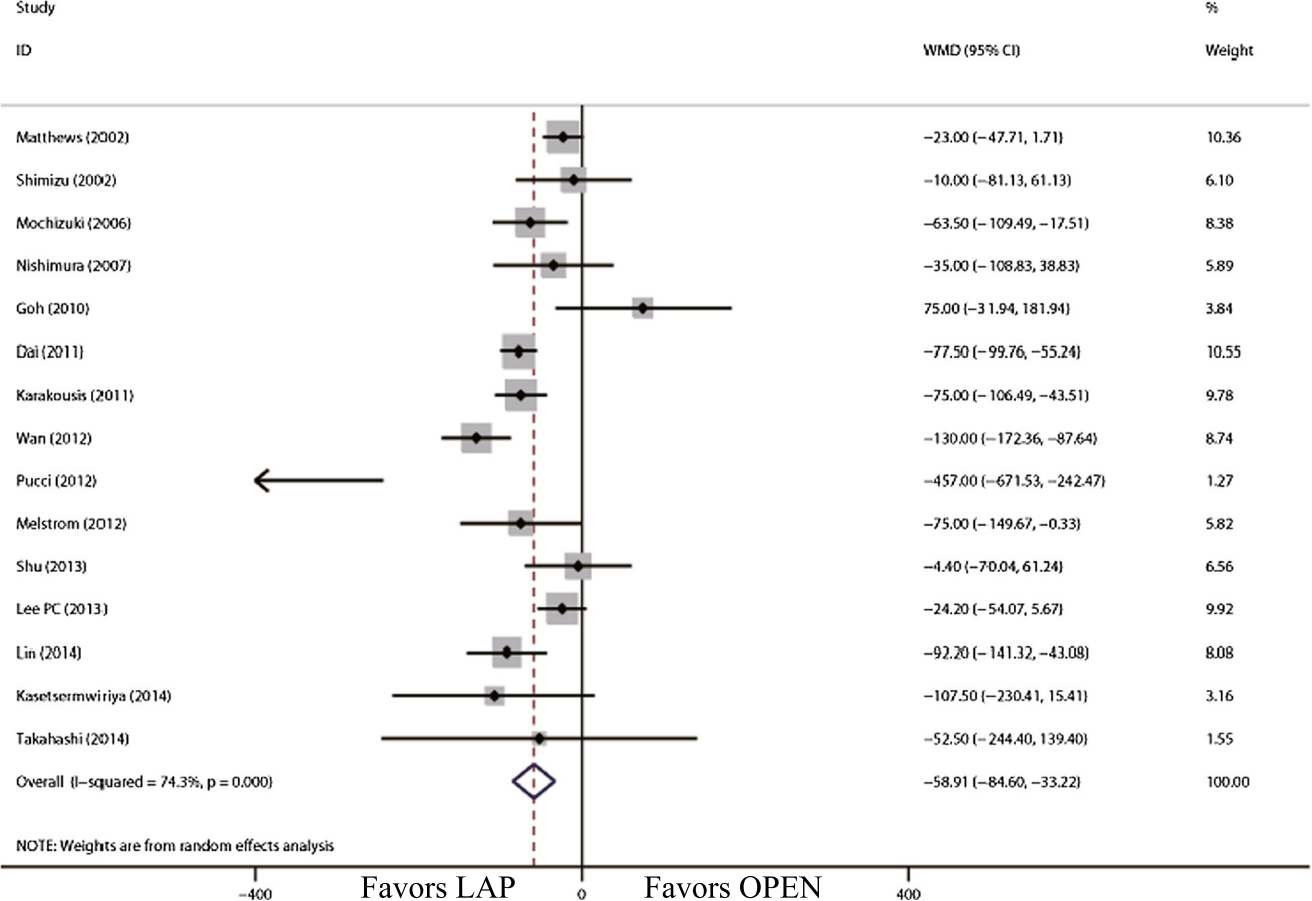
Meta-analysis of the pooled data: intraoperative blood loss (ml).

The tumor size for LAP was significantly smaller than that for OPEN (WMD = -0.98 cm; 95% CI, -1.36 to -0.60; *P* <0.01; Figure 2). The present analysis showed no statistically significant difference in the operation time of the two groups (WMD = -11.22 min; 95% confidence interval (CI), -28.66 to 6.23; *P* = 0.21; Figure 3). Intraoperative blood loss was significantly lower in the LAP compared with the OPEN group (WMD = -58.91 ml; 95% CI, -84.60 to -33.22 ml; *P* <0.01; Figure 4).

### Comparison of short-term postoperative outcomes

**Figure 5:**
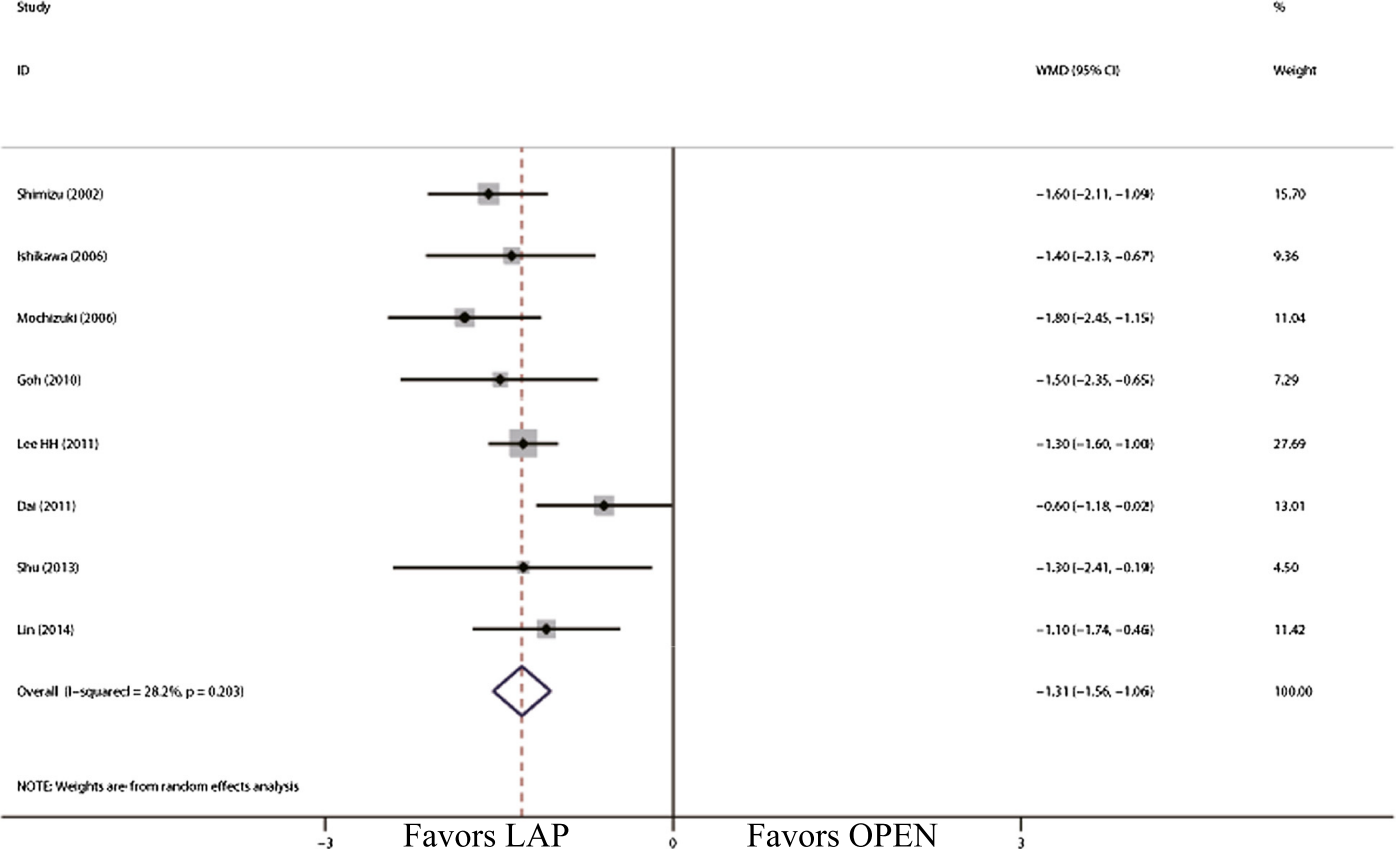
Meta-analysis of the pooled data: time to first flatus (days).

**Figure 6:**
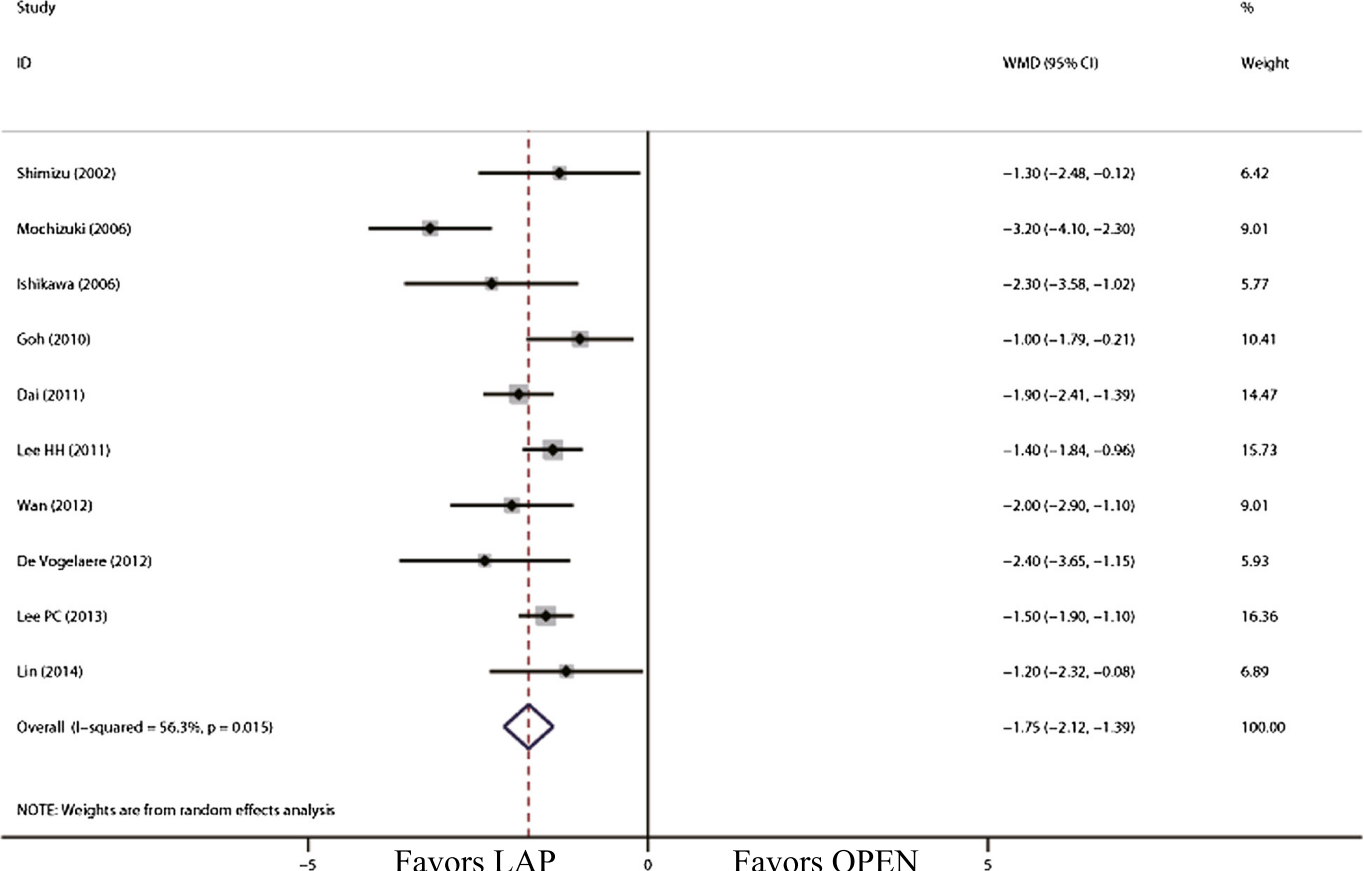
Meta-analysis of the pooled data: time to oral intake (days).

**Figure 7:**
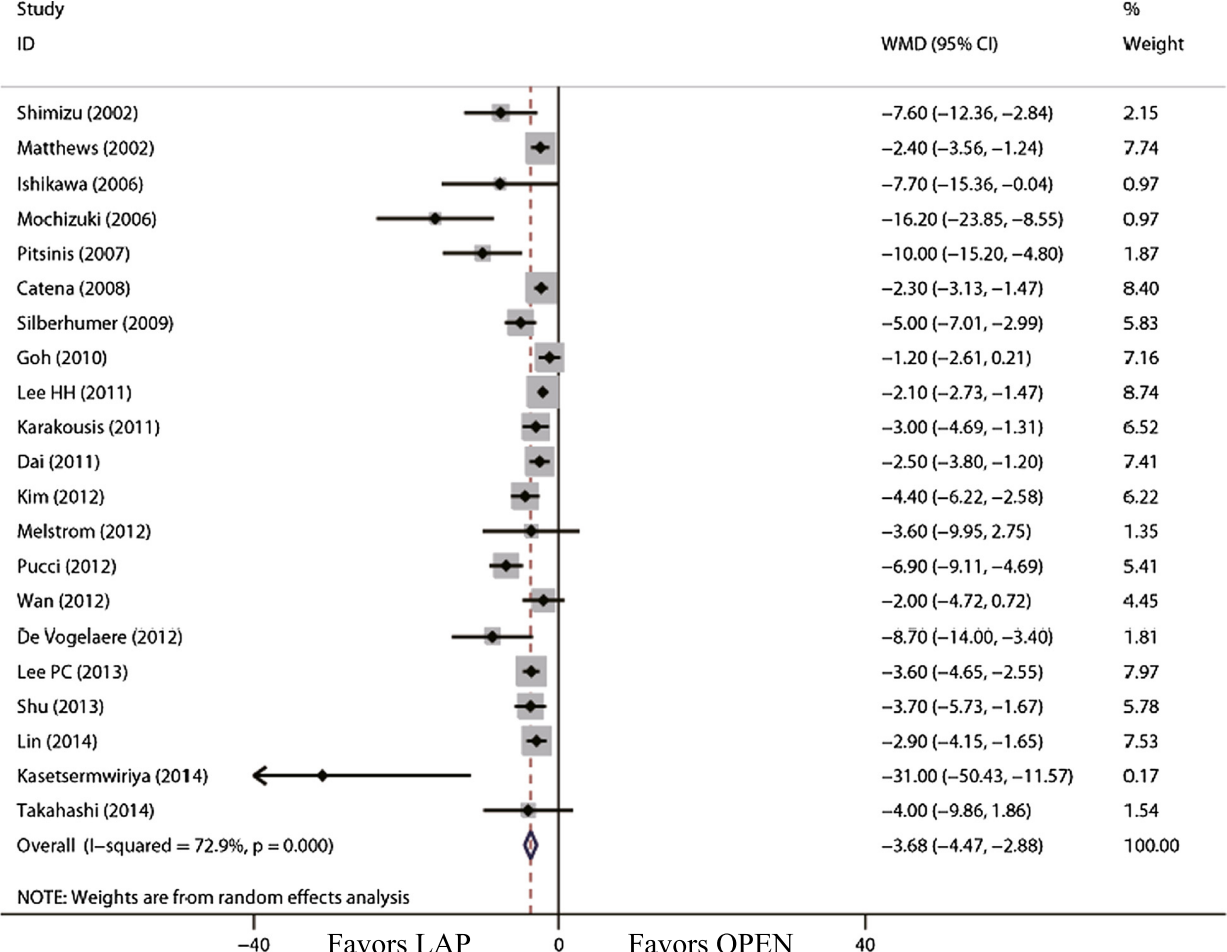
Meta-analysis of the pooled data: hospital stay (days).

**Figure 8:**
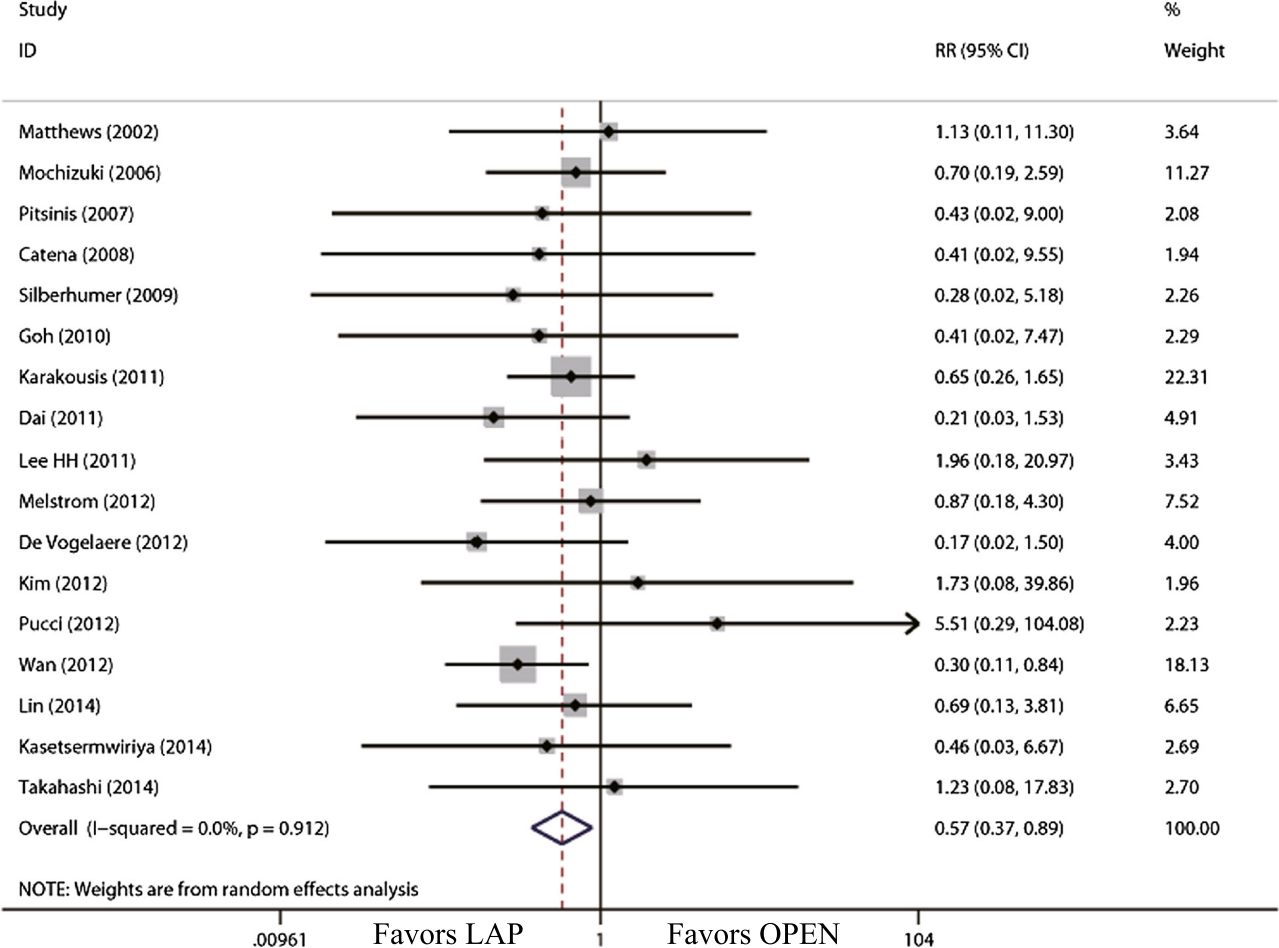
Meta-analysis of the pooled data: overall complications.

The outcomes also favored LAP in first flatus day (WMD = -1.31 d; 95% CI, -1.56 to -1.06; *P* <0.01; Figure 5) and first oral intake (WMD = -1.75 d; 95% CI, -2.12 to -1.39; *P* <0.01; Figure 6). Moreover, postoperative hospital day was 3.68 days shorter for LAP patients (WMD = -3.68 d; 95% CI, -4.47 to -2.88; *P* <0.01; Figure 7). With respect to the rate of overall postoperative complications, LAP is significantly superior to OPEN. The rate of overall postoperative complications was significantly lower for LAP (RR = 0.57; 95% CI, 0.37 to 0.89; *P* = 0.01; Figure 8). After further analysis, surgical complications were similar between the two groups (RR = 0.69; 95% CI, 0.37 to 1.29; *P* = 0.24). However, LAP was associated with a marginal reduction in systematic complications (RR = 0.57; 95% CI, 0.32 to 1.04; *P* = 0.07).

### Comparison of oncological outcomes

**Figure 9:**
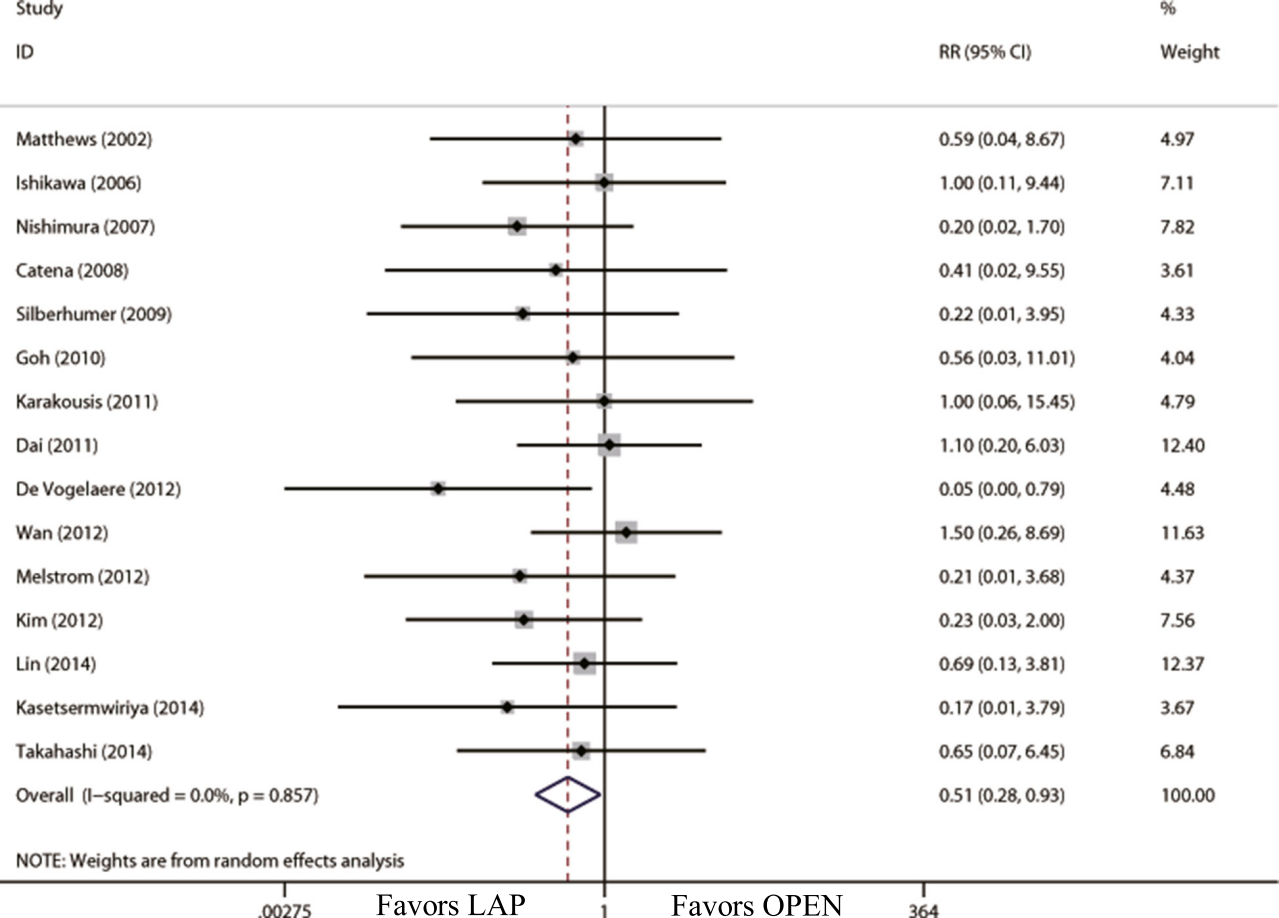
Meta-analysis of the pooled data: recurrences.

15 studies reported tumor recurrence [21,22,24,26-32,34,36,39-41]. The recurrence risk in LAP was 3.6% (14 out of 388) and 9.7% (38 out of 393) in OPEN, and patients who underwent LAP were less likely than the OPEN group to have recurrence (RR = 0.51; 95% CI, 0.28 to 0.93; *P* = 0.03; Figure 9). The available data about recurrence patterns and survival outcomes are summarized in Table 2.

**Table 2:** Summary of the available data about recurrence patterns and survival outcomes

**Case**	**Group**	**Risk**	**Recurrence**	**Survival**	**Case**	**Group**	**Risk**	**Recurrence**	**Survival**	**Case**	**Group**	**Risk**	**Recurrence**	**Survival**
[21]	LAP	High	NR	Dead	[26]	OPEN	NR	NR	Dead	[31]	OPEN	High	6 m Liver	52 m Alive^a^
[21]	OPEN	High	NR	14 m Dead	[27]	OPEN	High	Local	Dead^b^	[31]	OPEN	High	9 m Liver	75 m Alive^a^
[22]	LAP	High	Liver	32 m Dead	[27]	OPEN	High	Local	Alive^a^	[31]	OPEN	High	21 m Liver	16y Dead^a^
[22]	OPEN	High	Liver	9 m Dead	[27]	OPEN	High	Liver	Alive^a^	[36]	OPEN	High	52 m Peritoneal	Dead^a^
[22]	LAP	Low	Local	Alive	[27]	OPEN	High	Multiple	Alive^a^	[36]	OPEN	High	60 m Liver	Alive
[24]	LAP	Low	33 m Local	Alive	[29]	LAP	High	Peritoneal	4 y Alive	[36]	OPEN	High	6 m Colon	Dead^a^
[24]	OPEN	High	7 m Peritoneal	Alive	[29]	OPEN	Moderate	Liver	10 y Alive	[36]	LAP	High	31 m Stomach	Alive
[24]	OPEN	High	53 m Local	Alive	[31]	OPEN	High	4 m Liver	28 m Dead^a^	[36]	OPEN	High	15 Peritoneal	Dead^a^
[24]	OPEN	High	37 m Liver	Alive	[31]	OPEN	High	10 m Liver	Alive^a^	[39]	OPEN	High	11 m Liver	59 m Alive
[24]	OPEN	High	15 m Multiple^d^	Alive	[31]	OPEN	High	42 m Liver	46 m Dead^a,c^					

LAP, laparoscopic surgery; m: month; NR, not reported; OPEN, open surgery; y: year.

^a^treated with imatinib; ^b^due to cardiac insufficiency; ^c^due to lung cancer; ^d^included liver and local recurrence.

### Comparison of wedge resection

Comparison data of laparoscopic wedge resection and open wedge resection was available in eight studies[20,22,23,26,28,30,33,34]. The overall effects such as operation time, blood loss, time to flatus or oral intake, hospital stay, and complications remained unchanged. However, in this subgroup analysis, the recurrence risk in LAP was 5.4% (7 out of 130) and 5.5% (9 out of 165) in OPEN, and the difference was not significant (RR = 1.01; 95% CI, 0.39 to 2.63; *P* = 0.99). The outcomes of subgroup analysis for studies of wedge resection are summarized in Table 3.

**Table 3:** Pooled outcomes of subgroup analysis for studies of wedge resection

**Outcomes**	**Number of studies**	**Sample size**		**Heterogeneity (*P, I***^***2***^**)**	**Overall effect size**	**95% CI of overall effect**	***P***
		**LAP**	**OPEN**				
Operation time (min)	8	203	233	<0.001, 82%	WMD = 12.03	-8.03, 32.09	0.24
Blood loss (ml)	5	118	151	0.03, 64%	WMD = -48.29	-78.23, -18.36	<0.01
Time to first flatus (d)	6	119	144	0.10, 46%	WMD = -1.35	-1.66, -1.03	<0.01
Time to oral intake (d)	7	182	208	0.001, 73%	WMD = -1.67	-2.19, -1.15	<0.01
Hospital stay (d)	8	203	233	0.002, 68%	WMD = -2.53	-3.50, -1.57	<0.01
Overall complications	8	203	233	0.70, 0%	RR = 0.47	0.22, 1.01	0.05
Tumor size (cm)	8	203	233	0.10, 42%	WMD = -0.77	-1.23, -0.31	<0.01
Recurrence	5	130	165	0.95, 0%	RR = 1.01	0.39, 2.63	0.99

CI, confidence interval; LAP, laparoscopic surgery; OPEN, open surgery; RR, risk ratio; WMD, weighted mean difference.

### Subgroup analysis for studies with comparable tumor size or risk index

Thirteen studies qualified for a subgroup analysis for studies with comparable tumor size or risk index [20,21,23,24,26,29,30,33,34,37-40]. Like the subgroup analysis for wedge resection, outcomes other than tumor recurrence remained unchanged. The recurrence risk was similar between LAP and OPEN (RR = 0.66; 95% CI, 0.31 to 1.42; *P* = 0.29). The outcomes of subgroup analysis for studies with comparable tumor size or risk index are summarized in Table 4.

**Table 4:** Pooled outcomes of subgroup analysis for studies with comparable tumor size or risk index

**Outcomes**	**Number of studies**	**Sample size**		**Heterogeneity (*P, I***^***2***^**)**	**Overall effect size**	**95% CI of overall effect**	***P***
		**LAP**	**OPEN**				
Operation time (min)	13	371	377	<0.001, 81%	WMD = -1.06	-16.93, 14.81	0.90
Blood loss (ml)	11	300	302	<0.001, 71%	WMD = -58.20	-81.76, -34.65	<0.01
Time to first flatus (d)	6	129	142	0.09, 47%	WMD = -1.28	-1.60, -0.97	<0.01
Time to oral intake (d)	7	212	241	0.02, 61%	WMD = -1.77	-2.18, -1.35	<0.01
Hospital stay (d)	11	309	339	0.006, 60%	WMD = -2.87	-3.54, -2.21	<0.01
Overall complications	12	341	345	0.74, 0%	RR = 0.49	0.30, 0.81	<0.01
Tumor size (cm)	13	371	377	0.23, 21%	WMD = -0.57	-0.86, -0.29	<0.01
Recurrence	8	248	232	0.81, 0%	RR = 0.66	0.31, 1.42	0.29

CI, confidence interval; LAP, laparoscopic surgery; OPEN, open surgery; RR, risk ratio; WMD, weighted mean difference.

### Publication bias

**Figure 10:**
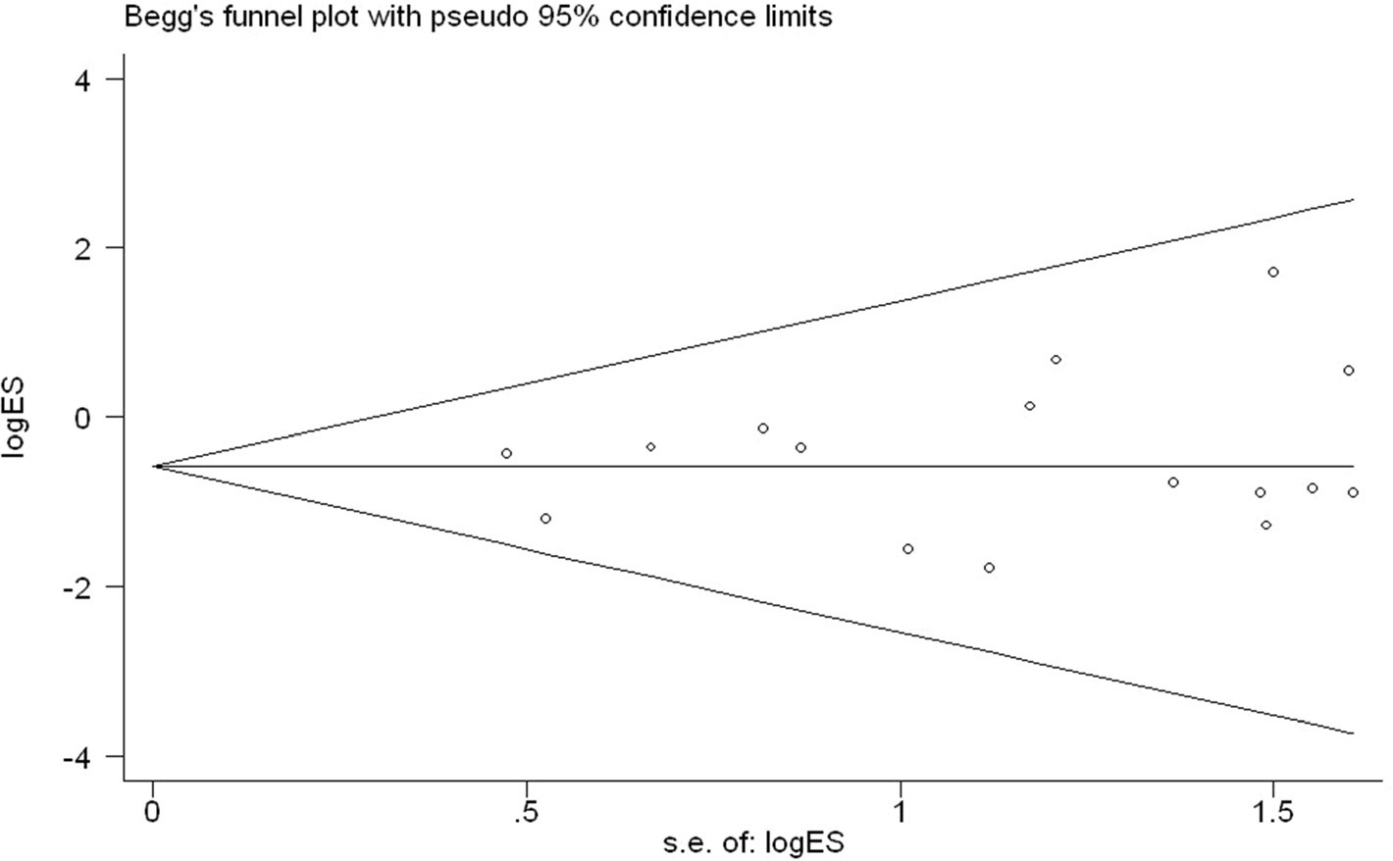
Funnel plot of the overall postoperative complications.

To test for publication bias, we used funnel plots and performed an Egger’s test based on the incidence of overall postoperative complications (Figure 10). The graphical funnel plot showed that none of the studies lay outside the 95% CI boundaries, and there was no evidence of publication bias.

## Discussion

GISTs, although rare, are the most common mesenchymal tumors arising in the wall of the gastrointestinal tract. Surgery remains the mainstay of definitive therapy for non-metastatic GISTs. Recent evidence suggests that prognosis is mainly based on tumor size and histological features rather than wide resection margins [3,42], which makes laparoscopic resection more popular for GIST treatment. Recently, some meta-analysises showed the superiority of LAP to OPEN [43,44]. With the development of the laparoscopic technique, several additional articles that compare LAP with OPEN have been published since that analysis [36-41]. Therefore, we performed this updated meta-analysis to broaden the current knowledge on the clinical value of LAP.

We failed to include randomized controlled trials (RCTs) in this study. Although RCTs are first choice for a high-quality of meta-analysis, there are some hurdles to overcome such as learning curve effects, ethical issues, and the relatively low incidence of GISTs during the conduction of a high-quality RCT to evaluate this new surgical approach. Therefore, we made a number of efforts to ensure convincing results from non-RCTs, including the use of NOS to assess the quality of the studies and exclude low-quality studies; conducting subgroup analysis for studies of wedge resection, comparable tumor size or risk index to minimize the selection bias; and using funnel plots and Egger’s test to detect publication bias.

Our pooled analysis demonstrated faster gastrointestinal recovery in LAP. Reduced use of analgesic drugs, milder acute inflammatory response, and earlier postoperative activities are considered to be the main reasons for earlier gastrointestinal recovery in this type of surgery. The meta-analysis demonstrated a reduced number of complications in the LAP versus OPEN group, which may have resulted from a reduction in systematic complications. It is conceivable that surgical complications were similar between groups because LAP, though less invasive, results in the same resection extent as OPEN. Decreased pulmonary infection, which is the most common systemic complication, could explain the reduced systemic complications in LAP. Pain after surgery was less serious in LAP than in OPEN, reflected by a shorter duration or lower dosage of analgesics. The pain caused by the large incision as well as the use of tension sutures and abdominal bandages after laparotomy would deter patients’ attempts to cough, expectorate and perform breathing exercise effectively, resulting in complications such as pulmonary infection [45]. Our study demonstrated the postoperative hospital stay was 3.6 days shorter for LAP patients, which reasonably results from faster gastrointestinal recovery and a reduced number of complications in LAP.

The present analysis demonstrated that the operative time in the LAP group was similar to OPEN, which is in contrast to many other types of gastrointestinal surgery [46-49]. This result was mainly based on two factors. Lymphadenectomy, which is complicated and time-consuming under laparoscopy, is not generally required in LAP. Time spent on the establishment of pneumoperitoneum and the closure of the trocar incision and mini-laparotomy is likely to be shorter than the opening and closing of a laparotomy. Additionally, most studies involving less operative time in the LAP group had a relatively larger sample size or were recently published [29,33-41], which might explain why the LAP appeared to be shorter than OPEN because of an accumulation of laparoscopic skills and the development of laparoscopic instruments.

Operative blood loss was shown in the pooled analysis to be lower in LAP. The reduced length of incision and the application of energy-dividing devices contribute to this reduction in blood loss. Moreover, the magnified view of laparoscopy allows for meticulous manipulation and reduction of injury. In our study, the asymmetric distribution of tumor size or extent of resection makes comparison of operative blood loss inherently flawed and at a high risk for confounding factors. So a subgroup analysis for studies with comparable tumor size or extent of resection was conducted, and less operative blood loss was still observed, which suggests that the technique of LAP itself might be the main reason for less operative blood loss.

Long-term survival remains critical for patients with GIST because of its malignant potential. Our study confirmed the safety of LAP for GISTs compared with OPEN. The postoperative recurrence in the LAP group was less than that of the OPEN group with statistical significance. However, the observed advantages of laparoscopy may be skewed by selection bias regarding tumor size. In several included studies, larger tumor size and higher risk classification were dominant in the OPEN group. According to the risk assessment classification [3], tumor size and mitotic index are two key factors on GISTs long-term outcomes. Thus, the studies with the same surgical approach (wedge resection) as well as those with comparable tumor size or risk classification were included in a subgroup analysis. The results of two subgroup analyses showed that the risk of postoperative recurrence in the LAP group was similar to the OPEN group. The increased experience of laparoscopic procedures, no touch of tumor and retrieving the tumor with an endobag [8,36] may have contributed to this result. In addition, we also observed that the common sites of postoperative recurrence of GISTs included liver metastasis, peritoneal metastasis and local recurrence. Most cases of recurrence or metastasis had a trend toward higher-risk profiles and no port metastasis was identified, which suggests tumor recurrence is not clearly related to the surgical approach [21,22,24,26,27,29,31,36,39].

There are several limitations to our studies that must be taken into account when considering the results. First, all of the studies included in this meta-analysis are non-RCTs, which could lead to substantial selection and observation bias. Second, despite the majority of studies analyzed focusing only on GISTs, some included studies had several cases of other types of gastric submucosal tumors, such as neurilemmomas and leiomyomas. Because the sample size of remaining studies was still small for definitive conclusions on the safety and effectiveness of LAP, we did not exclude the study. Although such a low number does not imply a significant bias, it still can lead to clinical heterogeneity. Third, although the funnel plot showed that publication bias is unlikely, clinicians must be aware of possible publication bias when using evidence in clinical practice. Also, the follow-up duration of cases in the meta-analysis is too short for the low-risk GISTs to have developed tumor recurrence, which may have an influence on the tumor recurrence rate, and more long-term follow-up studies are awaited.

## Conclusions

The current clinical evidence revealed that LAP is safe and feasible for the treatment of gastric GISTs in regards to short- and long-term outcomes. In selective patients, LAP is preferable compared with OPEN for its minimally invasive advantages. More well-designed RCTs or prospective cohort studies are awaited to adequately evaluate the status of laparoscopic resection for gastric GISTs.

## Abbreviations

CI: confidence interval; GISTs: gastrointestinal stromal tumors; LAP: laparoscopic surgery; NOS: Newcastle-Ottawa Quality Assessment Scale; OPEN: open surgery; RCTs: randomized controlled trials; RR: relative risk; WMD: weighted mean difference.

## Competing interests

The authors declare that they have no competing interests.

## Authors’ contributions

QLC and YP wrote the manuscript; JQC, DW and KC performed the literature review and conducted the analysis of pooled data; YPM proofread and revised the manuscript. All authors read and approved the final manuscript.

## References

[B1] Miettinen M Majidi M Lasota J Pathology and diagnostic criteria of gastrointestinal stromal tumors (GISTs): a review Eur J Cancer 2002 38 Suppl 5 S39 51 12528772 10.1016/s0959-8049(02)80602-5

[B2] Demetri GD von Mehren M Antonescu CR DeMatteo RP Ganjoo KN Maki RG Pisters PW Raut CP Riedel RF Schuetze S Sundar HM Trent JC Wayne J NCCN Task Force report: update on the management of patients with gastrointestinal stromal tumors J Natl Compr Canc Netw 2010 2 S1 41 quiz S42-44 20457867 10.6004/jnccn.2010.0116 PMC4103754

[B3] Fletcher CD Berman JJ Corless C Gorstein F Lasota J Longley BJ Miettinen M O’Leary TJ Remotti H Rubin BP Shmookler B Sobin LH Weiss SW Diagnosis of gastrointestinal stromal tumors: a consensus approach Hum Pathol 2002 33 459 465 12094370 10.1053/hupa.2002.123545

[B4] DeMatteo RP Lewis JJ Leung D Mudan SS Woodruff JM Brennan MF Two hundred gastrointestinal stromal tumors: recurrence patterns and prognostic factors for survival Ann Surg 2000 231 51 58 10636102 10.1097/00000658-200001000-00008 PMC1420965

[B5] Piso P Schlitt HJ Klempnauer J Stromal sarcoma of the stomach: therapeutic considerations Eur J Surg 2000 166 954 958 11152257 10.1080/110241500447128

[B6] Xu X Chen K Zhou W Zhang R Wang J Wu D Mou Y Laparoscopic transgastric resection of gastric submucosal tumors located near the esophagogastric junction J Gastrointest Surg 2013 17 1570 1575 23771749 10.1007/s11605-013-2241-2

[B7] Kang WM Yu JC Ma ZQ Zhao ZR Meng QB Ye X Laparoscopic-endoscopic cooperative surgery for gastric submucosal tumors World J Gastroenterol 2013 19 5720 5726 24039367 10.3748/wjg.v19.i34.5720 PMC3769911

[B8] Valle M Federici O Carboni F Carpano S Benedetti M Garofalo A Gastrointestinal stromal tumors of the stomach: the role of laparoscopic resection. Single-centre experience of 38 cases Surg Endosc 2014 28 1040 1047 24149857 10.1007/s00464-013-3255-2

[B9] Lee CH Hyun MH Kwon YJ Cho SI Park SS Deciding laparoscopic approaches for wedge resection in gastric submucosal tumors: a suggestive flow chart using three major determinants J Am Coll Surg 2012 215 831 840 22951033 10.1016/j.jamcollsurg.2012.07.009

[B10] Grobmyer SR Pieracci FM Allen PJ Brennan MF Jaques DP Defining morbidity after pancreaticoduodenectomy: use of a prospective complication grading system J Am Coll Surg 2007 204 356 364 17324768 10.1016/j.jamcollsurg.2006.11.017

[B11] Hozo SP Djulbegovic B Hozo I Estimating the mean and variance from the median, range, and the size of a sample BMC Med Res Methodol 2005 5 13 15840177 10.1186/1471-2288-5-13 PMC1097734

[B12] Higgins JP Thompson SG Deeks JJ Altman DG Measuring inconsistency in meta-analyses BMJ 2003 327 557 560 12958120 10.1136/bmj.327.7414.557 PMC192859

[B13] DerSimonian R Laird N Meta-analysis in clinical trials Control Clin Trials 1986 7 117 188 10.1016/0197-2456(86)90046-2 3802833

[B14] Basu S Balaji S Bennett DH Davies N Gastrointestinal stromal tumors (GIST) and laparoscopic resection Surg Endosc 2007 21 1685 1689 17661137 10.1007/s00464-007-9445-z

[B15] Chen YH Liu KH Yeh CN Hsu JT Liu YY Tsai CY Chiu CT Jan YY Yeh TS Laparoscopic resection of gastrointestinal stromal tumors: safe, efficient, and comparable oncologic outcomes J Laparoendosc Adv Surg Tech A 2012 22 7587 7563 10.1089/lap.2012.0115 22957924

[B16] Fisher SB Kim SC Kooby DA Cardona K Russell MC Delman KA Staley CA 3rd Maithel SK Gastrointestinal stromal tumors: a single institution experience of 176 surgical patients Am Surg 2013 79 657 665 23815996

[B17] Otani Y Furukawa T Yoshida M Saikawa Y Wada N Ueda M Kubota T Mukai M Kameyama K Sugino Y Kumai K Kitajima M Operative indications for relatively small (2-5 cm) gastrointestinal stromal tumor of the stomach based on analysis of 60 operated cases Surgery 2006 139 484 492 16627057 10.1016/j.surg.2005.08.011

[B18] Wu JM Yang CY Wang MY Wu MH Lin MT Gasless laparoscopy-assisted versus open resection for gastrointestinal stromal tumors of the upper stomach: preliminary results J Laparoendosc Adv Surg Tech A 2010 20 725 729 20969456 10.1089/lap.2010.0231

[B19] Bellorin O Kundel A Ni M Litong D Surgical management of gastrointestinal stromal tumors of the stomach JSLS 2014 18 46 49 24680142 10.4293/108680813X13693422522150 PMC3939341

[B20] Shimizu S Noshiro H Nagai E Uchiyama A Mizumoto K Tanaka M Laparoscopic wedge resection of gastric submucosal tumors Dig Surg 2002 19 169 173 12119518 10.1159/000064209

[B21] Matthews BD Walsh RM Kercher KW Sing RF Pratt BL Answini GA Heniford BT Laparoscopic vs open resection of gastric stromal tumors Surg Endosc 2002 16 803 807 11997826 10.1007/s00464-001-8319-z

[B22] Ishikawa K Inomata M Etoh T Shiromizu A Shiraishi N Arita T Kitano S Long-term outcome of laparoscopic wedge resection for gastric submucosal tumor compared with open wedge resection Surg Laparosc Endosc Percutan Tech 2006 16 82 85 16773006 10.1097/00129689-200604000-00005

[B23] Mochizuki Y Kodera Y Fujiwara M Ito S Yamamura Y Sawaki A Yamao K Kato T Laparoscopic wedge resection for gastrointestinal stromal tumors of the stomach: initial experience Surg Today 2006 36 341 347 16554991 10.1007/s00595-005-3164-7

[B24] Nishimura J Nakajima K Omori T Takahashi T Nishitani A Ito T Nishida T Surgical strategy for gastric gastrointestinal stromal tumors: laparoscopic vs. open resection Surg Endosc 2007 21 875 878 17180273 10.1007/s00464-006-9065-z

[B25] Pitsinis V Khan AZ Cranshaw I Allum WH: Single center experience of laparoscopic vs. open resection for gastrointestinal stromal tumors of the stomach. Hepatogastroenterology 2007 54 606 608 17523332

[B26] Catena F Di Battista M Fusaroli P Ansaloni L Di Scioscio V Santini D Pantaleo M Biasco G Caletti G Pinna A Laparoscopic treatment of gastric GIST: report of 21 cases and literature’s review J Gastrointest Surg 2008 12 561 568 18040747 10.1007/s11605-007-0416-4

[B27] Silberhumer GR Hufschmid M Wrba F Gyoeri G Schoppmann S Tribl B Wenzl E Prager G Laengle F Zacherl J Surgery for gastrointestinal stromal tumors of the stomach J Gastrointest Surg 2009 13 1213 1219 19357931 10.1007/s11605-009-0872-0

[B28] Goh BK Chow PK Chok AY Chan WH Chung YF Ong HS Wong WK Impact of the introduction of laparoscopic wedge resection as a surgical option for suspected small/medium-sized gastrointestinal stromal tumors of the stomach on perioperative and oncologic outcomes World J Surg 2010 34 1847 1852 20407770 10.1007/s00268-010-0590-5

[B29] Karakousis GC Singer S Zheng J Gonen M Coit D DeMatteo RP Strong VE Laparoscopic versus open gastric resections for primary gastrointestinal stromal tumors (GISTs): a size-matched comparison Ann Surg Oncol 2011 18 1599 1605 21207158 10.1245/s10434-010-1517-y PMC4986692

[B30] Dai QQ Ye ZY Zhang W Lv ZY Shao QS Sun YS Tao HQ Laparoscopic versus open wedge resection for gastrointestinal stromal tumors of the stomach: a clinical controlled study Chin J Gastrointest Surg 2011 14 603 605 21866452

[B31] De Vogelaere K Hoorens A Haentjens P Delvaux G Laparoscopic versus open resection of gastrointestinal stromal tumors of the stomach Surg Endosc 2013 27 1546 1554 23233005 10.1007/s00464-012-2622-8

[B32] Melstrom LG Phillips JD Bentrem DJ Wayne JD Laparoscopic versus open resection of gastric gastrointestinal stromal tumors Am J Clin Oncol 2012 35 451 454 21552096 10.1097/COC.0b013e31821954a7

[B33] Lee HH Hur H Jung H Park CH Jeon HM Song KY Laparoscopic wedge resection for gastric submucosal tumors: a size-location matched case-control study J Am Coll Surg 2011 212 195 199 21147003 10.1016/j.jamcollsurg.2010.10.008

[B34] Wan P Yan C Li C Yan M Zhu ZG Choices of surgical approaches for gastrointestinal stromal tumors of the stomach: laparoscopic versus open resection Dig Surg 2012 29 243 250 22846475 10.1159/000341497

[B35] Pucci MJ Berger AC Lim PW Chojnacki KA Rosato EL Palazzo F Laparoscopic approaches to gastric gastrointestinal stromal tumors: an institutional review of 57 cases Surg Endosc 2012 26 3509 3514 22684977 10.1007/s00464-012-2374-5

[B36] Kim KH Kim MC Jung GJ Kim SJ Jang JS Kwon HC Long term survival results for gastric GIST: is laparoscopic surgery for large gastric GIST feasible? World J Surg Oncol 2012 10 230 23114111 10.1186/1477-7819-10-230 PMC3517899

[B37] Shu ZB Sun LB Li JP Li YC Ding DY Laparoscopic versus open resection of gastric gastrointestinal stromal tumors Chin J Cancer Res 2013 25 175 182 23592898 10.3978/j.issn.1000-9604.2013.02.03 PMC3626992

[B38] Lee PC Lai PS Yang CY Chen CN Lai IR Lin MT A gasless laparoscopic technique of wide excision for gastric gastrointestinal stromal tumor versus open method World J Surg Oncol 2013 11 44 23433002 10.1186/1477-7819-11-44 PMC3598221

[B39] Kasetsermwiriya W Nagai E Nakata K Nagayoshi Y Shimizu S Tanaka M Laparoscopic surgery for gastric gastrointestinal stromal tumor is feasible irrespective of tumor size J Laparoendosc Adv Surg Tech A 2014 24 123 129 24625346 10.1089/lap.2013.0433

[B40] Lin J Huang C Zheng C Li P Xie J Wang J Lu J Laparoscopic versus open gastric resection for larger than 5 cm primary gastric gastrointestinal stromal tumors (GIST): a size-matched comparison Surg Endosc 2014 [Epub ahead of print] 10.1007/s00464-014-3506-x 24853837

[B41] Takahashi T Nakajima K Miyazaki Y Miyazaki Y Kurokawa Y Yamasaki M Miyata H Takiguchi S Nishida T Mori M Doki Y Surgical strategy for the gastric gastrointestinal stromal tumors (GISTs) larger than 5 cm: laparoscopic surgery is feasible, safe, and oncologically acceptable Surg Laparosc Endosc Percutan Tech 2014 [Epub ahead of print] 10.1097/SLE.0000000000000039 24752159

[B42] Miettinen M El-Rifai WHL Sobin L Lasota J Evaluation of malignancy and prognosis of gastrointestinal stromal tumors: a review Hum Pathol 2002 33 478 483 12094372 10.1053/hupa.2002.124123

[B43] Koh YX Chok AY Zheng HL Tan CS Chow PK Wong WK Goh BK A systematic review and meta-analysis comparing laparoscopic versus open gastric resections for gastrointestinal stromal tumors of the stomach Ann Surg Oncol 2013 20 3549 3560 23793362 10.1245/s10434-013-3051-1

[B44] Chen K Zhou YC Mou YP Xu XW Jin WW Ajoodhea H Systematic review and meta-analysis of safety and efficacy of laparoscopic resection for gastrointestinal stromal tumors of the stomach Surg Endosc 2014 [Epub ahead of print] 10.1007/s00464-014-3676-6 25005014

[B45] Ephgrave KS Kleiman-Wexler R Pfaller M Booth B Werkmeister L Young S Postoperative pneumonia: a prospective study of risk factors and morbidity Surgery 1993 114 815 819 discussion 819-821 8211699

[B46] Chen K Xu XW Zhang RC Pan Y Wu D Mou YP Systematic review and meta-analysis of laparoscopy-assisted and open total gastrectomy for gastric cancer World J Gastroenterol 2013 19 5365 5376 23983442 10.3748/wjg.v19.i32.5365 PMC3752573

[B47] Xie K Zhu YP Xu XW Chen K Yan JF Mou YP Laparoscopic distal pancreatectomy is as safe and feasible as open procedure: a meta-analysis World J Gastroenterol 2012 18 1959 1967 22563178 10.3748/wjg.v18.i16.1959 PMC3337573

[B48] Law WL Lee YM Choi HK Seto CL Ho JW Impact of laparoscopic resection for colorectal cancer on operative outcomes and survival Ann Surg 2007 245 1 7 17197957 10.1097/01.sla.0000218170.41992.23 PMC1867940

[B49] Chen K Mou YP Xu XW Cai JQ Wu D Pan Y Zhang RC Short-term surgical and long-term survival outcomes after laparoscopic distal gastrectomy with D_2_ lymphadenectomy for gastric cancer BMC Gastroenterol 2014 14 41 24568165 10.1186/1471-230X-14-41 PMC3939636

